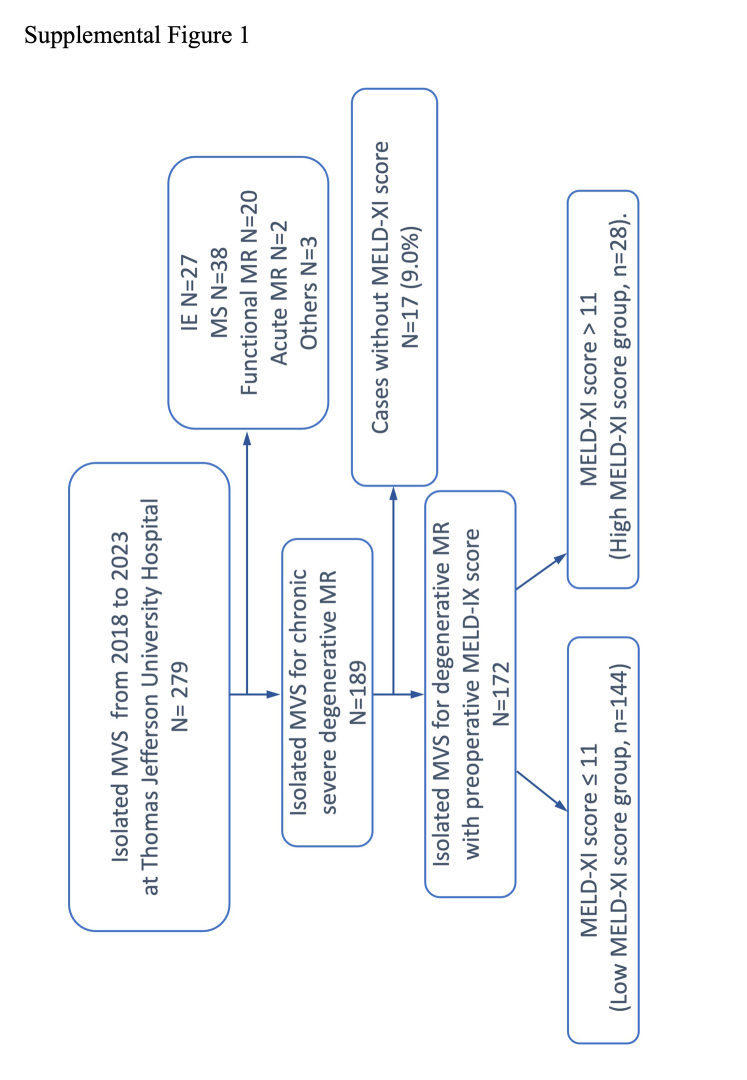# Preoperative MELD-XI Score and Risk of Heart Failure After Mitral Valve Surgery for Degenerative Mitral Regurgitation

**DOI:** 10.1016/j.atssr.2025.09.035

**Published:** 2025-11-04

**Authors:** Yuki Nakamura, Tomoki Sakata, Scott H. Koeneman, Keshava Rajagopal, Vakhtang Tchantchaleishvili, Konstadinos A. Plestis, John W. Entwistle, Joseph E. Bavaria, Rakesh M. Suri

**Affiliations:** 1 Division of Cardiac Surgery, Department of Surgery, Thomas Jefferson University, Philadelphia, Pennsylvania; 2 Division of Biostatistics and Bioinformatics, Thomas Jefferson University, Philadelphia, Pennsylvania; 3 Department of Cardiac Surgery, Corewell Health William Beaumont University Hospital, Royal Oak, Michigan

## Abstract

**Background:**

This study aimed to assess the association between the Model for End-Stage Liver Disease excluding international normalized ratio (MELD-XI) score and heart failure (HF) after isolated mitral valve surgery for degenerative mitral valve regurgitation (dMR).

**Methods:**

Patients undergoing mitral valve surgery for dMR were divided into groups of high MELD-XI score (>11, n = 28) and low MELD-XI score (≤11, n = 144). The primary end point was early HF events, defined as operative mortality, use of mechanical circulatory support, and prolonged use of inotropic agents after the surgery. The secondary end point was a composite outcome consisting of all-cause mortality and HF admission. The median follow-up period was 338 (70-789) days.

**Results:**

Thirty-day mortality rate was 0.7% in the low MELD-XI score group and 3.6% in the high MELD-XI score group (*P* = .737). The prolonged use of inotropic agents was significantly higher in the high MELD-XI score group than in the low MELD-XI score group (25.0% vs 6.9%; *P* = .015). Freedom from composite outcomes was significantly lower in the high MELD-XI score group than in the low MELD-XI score group (log-rank, *P* < .001). MELD-XI score (odds ratio, 1.10 [95% CI, 1.01-1.19]; *P* = .023) was identified as a significant risk factor of the early HF events. Multivariate Cox regression analysis found the MELD-XI score (hazard ratio, 1.07 [95% CI, 1.01-1.14]; *P* = .027) to be a significant risk factor for the composite outcome after surgery.

**Conclusions:**

In patients undergoing isolated mitral valve surgery for dMR, elevated preoperative MELD-XI score was associated with an increased risk of early HF events and the composite outcome.

**In Short**

▪ Preoperative elevated Model for End-Stage Liver Disease excluding international normalized ratio (MELD-XI) score was related to heart failure events early after mitral valve surgery for degenerative mitral regurgitation.
▪ Preoperative elevated MELD-XI score was associated with a composite outcome consisting of mortality and heart failure admission after mitral valve surgery for degenerative mitral regurgitation.

Whereas mitral valve surgery (MVS) for degenerative mitral valve regurgitation (dMR) is generally effective in improving symptoms and survival, heart failure (HF) may develop in a subset of patients postoperatively.^1^ Identifying patients at higher risk for development of HF after MVS is crucial for optimizing perioperative management and postsurgical surveillance. Several risk factors have been proposed, including preoperative New York Heart Association grade, atrial fibrillation, pulmonary hypertension, and impaired left ventricular function.^2^ However, there is a need for more comprehensive risk stratification tools that integrate multiple clinical and biochemical parameters.

The Model for End-Stage Liver Disease excluding international normalized ratio (INR) score (MELD-XI) is one of the scoring models that have been widely used for assessment of renal and hepatic function.^3^ This score is calculated by bilirubin and creatinine levels; the INR is also required for calculating the traditional MELD score in addition to bilirubin and creatinine. Previous reports showed the prognostic value of the MELD-XI score in patients with HF.^4^ However, the utility of the preoperative MELD-XI score in predicting HF after MVS for dMR has not been examined. This study aimed to assess the association between the MELD-XI score and HF outcomes after isolated MVS for dMR.

## Patients and Methods

### Study Population

We identified 279 patients who underwent isolated MVS between January 2018 and December 2023; 90 with non-dMR causes and 17 (9.0%) without preoperative MELD-XI scores because of missing laboratory values were excluded. The final cohort comprised 172 patients with dMR and available MELD-XI scores (Supplemental Figure). Median follow-up was 338 days (interquartile range, 70-789 days).

### Assessment of Hepatorenal Failure

Hepatorenal function was assessed by MELD-XI:

MELD−XI=5.11×ln(bilirubin[mg/dL])+11.76×ln(creatinine[mg/dL])+9.44

Based on prior reports,^4^^,^^5^ a score >11 defined high MELD-XI. There were 28 patients with high MELD-XI scores and 144 patients with low MELD-XI scores.

### End Points

Primary end points were early HF events (30-day mortality, mechanical circulatory support, or use of inotropic agents ≥48 hours) and late composite outcomes of all-cause mortality and HF readmission after MVS.

### Statistical Analysis

Normally distributed variables were expressed as mean ± SD and compared with the *t*-test. Nonnormally distributed variables were reported as median (interquartile range) and compared with the Wilcoxon test. Categorical variables were presented as counts (percentage) and compared with the *χ*^2^ test. Logistic regression identified factors for early outcomes and Cox regression for late composite outcomes. Variables with *P* < .05 in univariable analysis entered multivariable models. Kaplan-Meier curves for survival, freedom from HF admission, and composite outcomes were stratified by high or low MELD-XI score and compared with the log-rank test. Statistical significance was set at *P* < .05. Analyses were performed with EZR version 1.52 (Saitama Medical Center, Jichi Medical University), a graphical interface for R version 4.0.2 (R Foundation for Statistical Computing).

## Results

### Patient Characteristics and Surgical Variables

Patients with high MELD-XI score were significantly more likely to be older (age, 68 ± 11 years vs 63 ± 10 years; *P* = .012) and to have moderate to severe chronic obstructive pulmonary disease (5 [17.9%] vs 9 [6.3%]; *P* = .013), preoperative atrial fibrillation (14 [50.0%] vs 41 [28.5%]; *P* = .044), and larger preoperative left atrium diameter on echocardiography (49 [45-57] mm vs 43 [39-49] mm; *P* = .002; Table 1).

**Table 1:** Baseline Patient Characteristics

Characteristic	Low MELD-XI Score Group (n = 144)	High MELD-XI Score Group (n = 28)	*P* Value
Age, y	63 ± 10	68 ± 11	.012
Male	82 (56.9)	21 (75.0)	.116
Body mass index, kg/m^2^	26.9 (23.3-29.7)	26.2 (23.1-29.7)	.477
Smoking	64 (44.4)	11 (39.3)	.885
Diabetes mellitus	16 (11.1)	2 (7.1)	.741
Hypertension	98 (68.1)	22 (78.6)	.377
COPD ≥ moderate	9 (6.3)	5 (17.9)	.013
Cerebrovascular disease	13 (9.0)	4 (14.3)	.612
Heart failure	29 (20.1)	10 (35.7)	.120
Atrial fibrillation	41 (28.5)	14 (50.0)	.044
Laboratory data			
White blood cell count, ×10^3^/μL	6.7 (5.7-7.7)	6.7 (5.7-7.6)	.970
Hemoglobin, g/dL	13.8 (12.9-14.7)	13.8 (11.2-15.1)	.674
Hematocrit, %	41.7 (38.9-44.2)	41.0 (35.9-46.4)	.876
Platelet, ×10^4^/μL	21.1 (18.1-25.0)	18.3 (16.0-22.8)	.027
Albumin, mg/dL	4.5 (4.2-4.6)	4.1 (3.9-4.5)	.010
Creatinine, mg/dL	0.91 (0.80-1.04)	1.35 (1.16-1.70)	<.001
Total bilirubin, mg/dL	0.50 (0.40-0.70)	1.15 (0.58-1.60)	<.001
Echocardiogram			
LVDd, mm	53 (48-57)	54 (49-57)	.433
LVDs, mm	34 (30-37)	35 (30-37)	.816
LVEF, %	63 (60-65)	64 (58-65)	.474
IVSD, mm	10 (9-12)	10 (9-11)	.612
PWD, mm	10 (9-11)	11 (10-11)	.342
Left atrium diameter, mm	43 (39-49)	49 (45-57)	.002
Right atrium pressure, mm Hg	3 (3-5)	3 (3-6)	.520
Estimated sPAP, mm Hg	32 (25-42)	36 (28-44)	.506
TR grade ≥ moderate	17 (11.8)	6 (21.4)	.287
Right ventricular dysfunction	7 (4.9)	0 (0.0)	NA
STS score, %	0.57 (0.29-0.91)	1.89 (0.71-2.88)	<.001
Operative factors			
Operation time, min	274 (247-315)	278 (239-302)	.762
Cardiopulmonary bypass time, min	121 (101-139)	114 (98-126)	.247
Aortic cross-clamping time, min	76 (62-91)	71 (60-82)	.201
Mitral valve repair	119 (82.6)	20 (71.4)	.191
Mitral valve replacement	25 (17.4)	8 (28.6)	

Data are presented as mean ± SD or median with interquartile ranges for continuous variables and counts (percentages) for categorical variables.

COPD, chronic obstructive pulmonary disease; IVSD, interventricular septum diameter; LVDd, left ventricular diameter at end-diastole; LVDs, left ventricular diameter at end-systole; LVEF, left ventricular ejection fraction; MELD-XI, Model for End-Stage Liver Disease excluding international normalized ratio; NA, not applicable; PWD, posterior left ventricular wall diameter; sPAP, systolic pulmonary artery pressure; STS, The Society of Thoracic Surgeons; TR, tricuspid regurgitation.

### Postoperative Outcomes

Early outcomes are summarized in the Supplemental Table. The 30-day mortality rate was 0.7% in the low MELD-XI score group and 3.6% in the high MELD-XI score group (*P* = .737). The frequencies of prolonged dependence on inotropic support were significantly higher in the high MELD-XI score group than in the low MELD-XI score group (7 [25.0%] vs 10 [6.9%]; *P* = .015). Freedom from the composite outcomes of all-cause mortality and HF readmission were significantly lower in the high MELD-XI score group than in the low MELD-XI score group (log-rank, *P* = .002; Figure).

**Figure:**
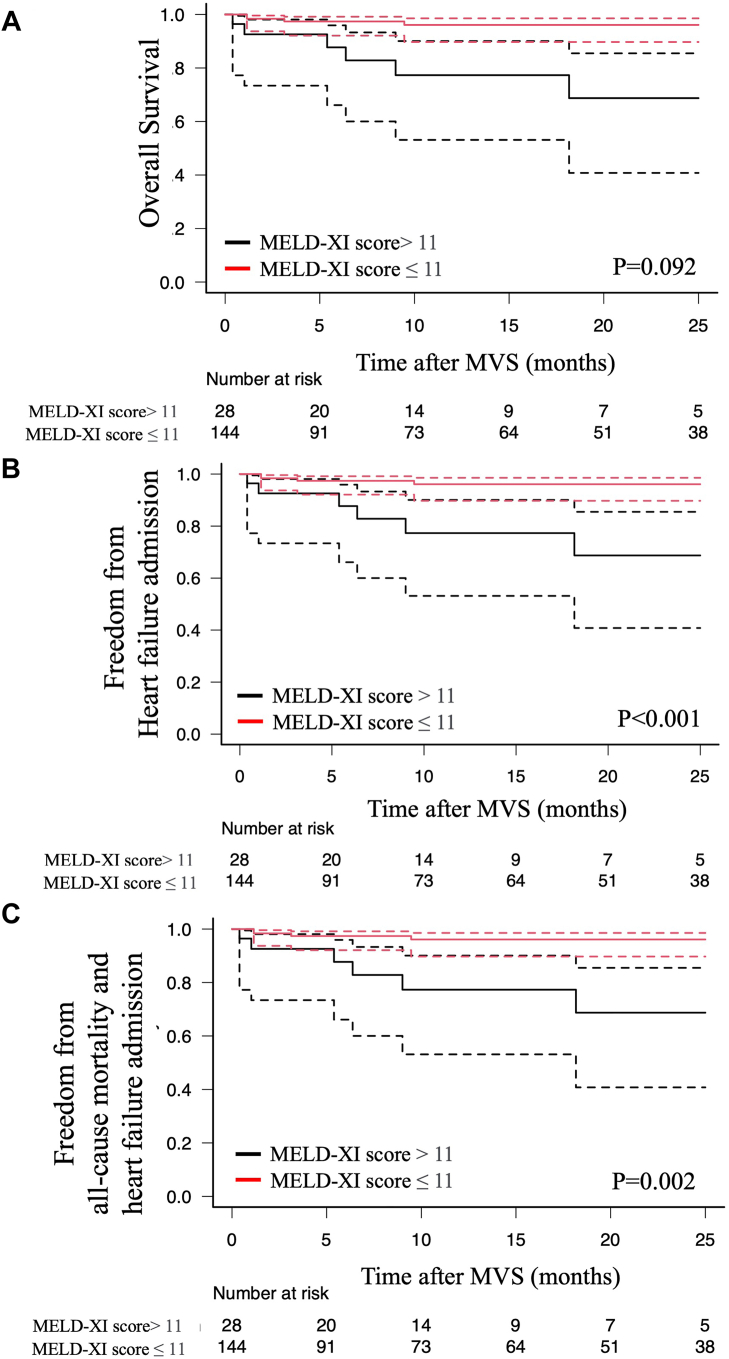
Kaplan-Meier curve showing (A) overall survival, (B) freedom from heart failure admission, and (C) freedom from all-cause mortality and heart failure admission. (MELD-XI, Model for End-Stage Liver Disease excluding international normalized ratio; MVS, mitral valve surgery.)

### Association Between MELD-XI Score and Clinical Outcomes

Logistic regression analysis revealed elevated MELD-XI score (odds ratio, 1.10 [95% CI, 1.01-1.19]; *P* = .023) to be a significant factor for the early HF event after MVS. Multivariate Cox regression analysis demonstrated elevated MELD-XI score (hazard ratio, 1.07 [95% CI, 1.01-1.14]; *P* = .027) to be a significant risk factor for the late composite outcomes after MVS (Table 2).

**Table 2:** Risk Factors for Early and Late Heart Failure–Related Outcomes

Logistic regression analysis for postoperative early heart failure events				
Factors	Univariable		Multivariable	
	OR (95% CI)	*P* Value	OR (95% CI)	*P* Value
Age	1.01 (0.96-1.05)	.784		
Male	1.05 (0.43-2.58)	.917		
Diabetes mellitus	2.91 (0.93-9.11)	.067		
Hypertension	3.27 (0.93-11.50)	.066		
COPD ≥ moderate	1.41 (0.79-2.52)	.245		
Cerebrovascular disease	2.20 (0.65-7.45)	.204		
Preoperative heart failure	2.55 (1.01-6.45)	.048	1.68 (0.60-4.65)	.322
Preoperative atrial fibrillation	2.19 (0.90-5.33)	.085		
LVDd	1.01 (0.95-1.07)	.833		
LVDs	1.03 (0.97-1.09)	.364		
LVEF	0.96 (0.91-1.02)	.158		
Left atrium diameter	1.04 (1.00-1.09)	.075		
Right atrium pressure	0.97 (0.83-1.13)	.676		
Estimated sPAP	1.02 (0.99-1.06)	.127		
Preoperative TR grade	1.33 (0.77-2.32)	.307		
Right ventricular dysfunction	1.08 (0.12-9.43)	.942		
MELD.XI score	1.11 (1.03-1.20)	.009	1.10 (1.01-1.19)	.0232
CPB time	1.01 (0.99-1.02)	.430		
AXC time	1.01 (1.00-1.03)	.103		
MVR	2.65 (1.01-6.91)	.047	2.38 (0.86-6.63)	.0959

Cox regression analysis for survival and heart failure				
Factors	Univariable		Multivariable	
	HR (95% CI)	*P* Value	HR (95% CI)	*P* Value
Age	1.04 (0.99-1.09)	.090		
Male	1.06 (0.4-2.79)	.902		
Body mass index	1.03 (0.96-1.1)	.423		
Smoking	1.02 (0.39-2.66)	.963		
Diabetes mellitus	1.20 (0.16-9.08)	.862		
Hypertension	1.74 (0.5-6.06)	.387		
COPD ≥ moderate	1.33 (0.72-2.47)	.362		
Cerebrovascular disease	1.29 (0.37-4.53)	.693		
Preoperative heart failure	1.91 (0.7-5.16)	.204		
Preoperative atrial fibrillation	1.06 (0.39-2.88)	.903		
LVDd	1.04 (0.96-1.12)	.342		
LVDs	1.04 (0.98-1.1)	.225		
LVEF	0.96 (0.91-1.02)	.211		
Left atrium diameter	1.03 (0.98-1.08)	.214		
Right atrium pressure	1.00 (0.86-1.17)	.961		
Estimated sPAP	1.02 (1-1.05)	.091		
Preoperative TR grade	1.88 (1.1-3.19)	.020	1.73 (0.99-3.02)	.053
Right ventricular dysfunction	2.07 (0.47-9.11)	.338		
MELD-XI score	1.09 (1.02-1.16)	.008	1.07 (1.01-1.14)	.0265
CPB time	0.99 (0.98-1.01)	.41		
AXC time	0.98 (0.95-1)	.097		
MVR	1.41 (0.46-4.34)	.550		

AXC, aortic cross-clamping; COPD, chronic obstructive pulmonary disease; CPB, cardiopulmonary bypass; HR, hazard ratio; LVDd, left ventricular diameter at end-diastole; LVDs, left ventricular diameter at end-systole; LVEF, left ventricular ejection fraction; MELD-XI, Model for End-Stage Liver Disease excluding international normalized ratio; MVR, mitral valve replacement; OR, odds ratio; sPAP, systolic pulmonary artery pressure; TR, tricuspid regurgitation.

## Comment

The main findings of this study were that elevated preoperative MELD-XI score is significantly associated with both the early combined HF events and the composite outcome of all-cause mortality and HF admission after MVS.

Originally developed for cirrhotic patients with anticoagulant therapy waiting for liver transplant, the MELD-XI score is commonly used for assessing hepatorenal function, particularly in patients with advanced liver dysfunction. Its prognostic value is also reported in patients with HF.^4^^,^^6^ The pathologic relationship between hepatorenal function and HF can be explained by reduced cardiac output and elevated cardiac filling pressures that cause the low renal and hepatic blood flow and the venous hypertension and may lead to worsening renal and hepatic function.^7^ It has been reported that a greater preoperative MELD-XI score is associated with higher early mortality in patients undergoing tricuspid valve surgery.^8^ The MELD-XI score was also reported to be a useful risk stratification tool for clinical outcomes including HF hospitalization after transcatheter mitral valve repair.^9^ Our study revealed increased preoperative MELD-XI score in patients with dMR to be associated with both early and late HF events after MVS. These findings may suggest that the hemodynamic effects of dMR associated with an elevated MELD-XI score affect clinical outcomes after MVS. The relationship between hemodynamic and physiologic factors warrants further investigation.

In this study, we hypothesized that preoperative hepatorenal assessment with the MELD-XI score could identify patients at risk for early and late HF events after MVS for dMR. Although The Society of Thoracic Surgeons and EuroSCORE II scores have been established for predicting mortality and morbidity early after cardiac surgery, they do not account for liver function, a well-known surgical risk factor influenced by hemodynamic abnormalities. The traditional MELD score, incorporating bilirubin, creatinine, and INR, has been reported to be a predictor of early outcomes after cardiac surgery.^10^ Given the frequent coexistence of atrial arrhythmias in patients with dMR, we posit that the traditional MELD score may be overestimated because of higher INR caused by preoperative anticoagulation therapy with vitamin K antagonists and may not be the ideal score to evaluate the hepatorenal function in such a cohort. In contrast, the MELD-XI score, calculated only with bilirubin and creatinine levels, is unaffected by preoperative anticoagulation and might serve as a precise and simple tool for hepatorenal assessment and risk stratification focused on postoperative HF in patients undergoing surgical correction of dMR, complementing The Society of Thoracic Surgeons and EuroSCORE II scores. The Child-Pugh classification has long been used to assess liver cirrhosis; however, its value in predicting postoperative HF is uncertain. It incorporates qualitative factors such as encephalopathy and ascites, making it less objective than MELD scores. HF risk assessment may benefit from evaluating multiple organ systems that are not fully captured by the Child-Pugh classification. Moreover, evidence for Child-Pugh classification is limited, whereas the association between MELD scores and HF outcomes has been reported.^4^^,^^6^^,^^9^ The MELD-XI score may provide a more objective and precise tool for perioperative HF risk assessment. Further studies are needed to validate this and to develop a comprehensive risk model in this cohort.

Our study has limitations. First, it was a single-center retrospective analysis. Second, 17 patients (9.0%) without preoperative MELD-XI scores because of missing laboratory values were excluded. In conclusion, an elevated preoperative MELD-XI score was associated with early HF events and the composite outcome of all-cause mortality and HF admission after MVS. The MELD-XI score may have potential value in identifying patients who could benefit from more intensive perioperative HF management and late HF surveillance; however, larger prospective studies are warranted to clarify its clinical relevance and practical implications.

## Figures and Tables

**Supplementary Figure 1:**