# Supplementary material for: Preoperative MELD-XI Score and Risk of Heart Failure After Mitral Valve Surgery for Degenerative Mitral Regurgitation
Source: Ann Thorac Surg Short Rep. 2025 Nov 4;4(2):607–12. doi: 10.1016/j.atssr.2025.09.035 (PMC13245306; doi:10.1016/j.atssr.2025.09.035)
Supplement: Supplementary Table 1 and Supplementary Figure 1 legend [file mmc1.docx]

Supplemental Materials

Supplemental Table 1. Early outcomes

|  | Low MELD-XI group (n=144) | High MELD-XI group (n=28) | P-value |
| --- | --- | --- | --- |
| 30 days mortality, n (%) | 1 ( 0.7) | 1 (3.6) | 0.737 |
| Bleeding, n (%) | 6 ( 4.2) | 0 (0.0) | NA |
| Stroke, n (%) | 4 ( 2.8) | 0 (0.0) | NA |
| Prolonged ventilation, n (%) | 8 (5.6) | 6 (21.4) | 0.015 |
| ICU stay (hours) | 44.5 (23.1-75.1) | 51.0 (26.6- 97.2) | 0.329 |
| Pneumonia, n (%) | 2 (1.4) | 1 (3.6) | 0.985 |
| Surgical site infection, n (%) | 0 (0.0) | 0 (0.0) | NA |
| Renal failure, n (%) | 5 (3.5) | 1 (3.6) | 1.000 |
| Pacemaker implantation, n (%) | 4 ( 2.8) | 3 ( 10.7) | 0.158 |
| Mechanical circulatory support, n (%) | 2 (1.4) | 1 (3.6) | 0.985 |
| Prolonged inotropes usage, n (%) | 10 (6.9) | 7 (25.0) | 0.015 |

Data are presented as the median with interquartile ranges for continuous variables and counts (percentages) for categorical variables.

ICU, intensive care unit; MELD-XI, model for end-stage liver disease excluding international normalized ratio.

**Supplemental Figure legends**

Supplemental Figure 1. Flow chart of patient selection

IE, infective endocarditis; MELD-XI, model for end-stage liver disease excluding international normalized ratio; MR; mitral valve regurgitation; MS, mitral stenosis
